# Optimizing the electron transport chain to sustainably improve photosynthesis

**DOI:** 10.1093/plphys/kiad490

**Published:** 2023-09-06

**Authors:** Lianhong Gu

**Affiliations:** Environmental Sciences Division and Climate Change Science Institute, Oak Ridge National Laboratory, Oak Ridge, TN 37831, USA

## Abstract

Genetically improving photosynthesis is a key strategy to boosting crop production to meet the rising demand for food and fuel by a rapidly growing global population in a warming climate. Many components of the photosynthetic apparatus have been targeted for genetic modification for improving photosynthesis. Successful translation of these modifications into increased plant productivity in fluctuating environments will depend on whether the electron transport chain (ETC) can support the increased electron transport rate without risking overreduction and photodamage. At present atmospheric conditions, the ETC appears suboptimal and will likely need to be modified to support proposed photosynthetic improvements and to maintain energy balance. Here, I derive photochemical equations to quantify the transport capacity and the corresponding reduction level based on the kinetics of redox reactions along the ETC. Using these theoretical equations and measurements from diverse C_3_/C_4_ species across environments, I identify several strategies that can simultaneously increase the transport capacity and decrease the reduction level of the ETC. These strategies include increasing the abundances of reaction centers, cytochrome b_6_f complexes, and mobile electron carriers, improving their redox kinetics, and decreasing the fraction of secondary quinone–nonreducing photosystem II reaction centers. I also shed light on several previously unexplained experimental findings regarding the physiological impacts of the abundances of the cytochrome b_6_f complex and plastoquinone. The model developed, and the insights generated from it facilitate the development of sustainable photosynthetic systems for greater crop yields.

## Teaser

Electron carriers and protein complexes along the electron transport chain can be optimized to increase photosynthesis without inducing oxidative stresses to plants in fluctuating environments.

## Introduction

Plant fitness in wild environments is defined primarily by reproductivity and survivability. These fitness traits do not generally include high yields of seeds or plant parts edible by animals including humans. As evolution selects plant species for fitness, aspects of a plant species of particular interest to humans such as productivity can be left out. The Green Revolution has greatly improved agricultural productivity, but globally, crop yields apparently have plateaued, signaling that the Green Revolution may have run its course (Zhu et al. 2010). Enhancing photosynthesis through bioengineered genetic modification of components of the photosynthetic machinery is considered an important pathway to overcoming the yield plateau and meeting the rising demand for food and fuel by a rapidly growing global population in a warming climate (Ort et al. 2015; Bailey-Serres et al. 2019; Burgess et al. 2023). Traditionally, photosynthesis is often separated into the light and biochemical carbon reactions (Buchanan 2016). For discussing the bioengineering of photosynthesis, it is useful to further separate the light reactions into the photophysical and photochemical reactions as these 2 types of reactions are conducted by spatially and temporally separated subsystems that operate at different time scales and follow different laws (Gu et al. 2023). The photophysical reactions cover the stage of light harvesting and excitation energy transfer to different dissipation pathways including the reaction centers of photosystems while the photochemical reactions follow the photophysical reactions and cover the stage of water splitting, electron transport, proton translocation, and the production of ATP and NADPH which are then utilized in the subsequent biochemical reactions of the Calvin–Benson cycle. Photosynthesis can be potentially enhanced by improving efficiencies in either the photophysical, photochemical, or biochemical reactions.

The photophysical model of Gu et al. (2019) provides a succinct framework for analyzing how the photophysical reactions can be modified such that more energy harvested by the antenna complexes is allocated to the photochemical pathway for photosynthesis. The partitioning of leaf-absorbed solar energy among different dissipation ways is governed by the principle of energy conservation. Assuming the lake model of photosynthetic unit connectivity (Kramer et al. 2004; Blankenship 2021), the linear electron transport (LET) rate (*J*_PSII_) from photosystem II (PSII) to photosystem I (PSI) is determined by the photosynthetic photon flux density (PPFD) according to the following photophysical equation of photosynthesis:


(1) $$J_{\mathrm{PSII}}=\frac{\beta \alpha \mathrm{PPFD}}{\frac{1+\mathrm{NPQ}}{q}\times \frac{1-\Phi_{\mathrm{PSIImax}}}{\Phi_{\mathrm{PSIImax}}}+1}.$$

Table 1 defines major symbols. Here, *α* is the leaf absorptance in photosynthetically active radiation, *β* is the fraction of absorbed photons allocated to PSII, NPQ is the parameter of nonphotochemical quenching (NPQ), *q* is the fraction of open PSII reaction centers under the lake model, and $\Phi_{\mathrm{PSIImax}}$ is the maximum photochemical quantum yield of PSII. Eq 1 shows that *J*_PSII_ per unit of PPFD can be potentially increased by increasing *α* and *β* or by decreasing $\frac{1+\mathrm{NPQ}}{q}$ and $\frac{1-\Phi_{\mathrm{PSIImax}}}{\Phi_{\mathrm{PSIImax}}}$. Among these potential options, increasing *β* is probably not going to achieve the goal of improving overall photosynthetic efficiency because increasing energy allocation to PSII would be at the expense of energy allocation to PSI, and yet, the balance between the 2 photosystems is necessary for photosynthetic electron transport (Gu et al. 2022). It has been observed that unstressed, healthy, dark-adapted leaves have remarkably similar maximum photochemical quantum yield of PSII at a value of about 0.83 across plant species (Björkman and Demmig 1987; Johnson et al. 1993). This value is smaller than that of PSI, which is about 0.94 to 0.98 and close to perfect (Hogewoning et al. 2012). Therefore, it may be possible to engineer a PSII with a higher $\Phi_{\mathrm{PSIImax}}$ to decrease $\frac{1-\Phi_{\mathrm{PSIImax}}}{\Phi_{\mathrm{PSIImax}}}$, which characterizes the intrinsic photochemical inefficiency of PSII. Since the difference in photochemical efficiency between the 2 photosystems is quite large (∼15%), improving photochemical efficiency of PSII should be a worthwhile effort.

**Table 1.** List of frequently used symbols and abbreviations

Symbol	Definition	Units
*a*_q_	The redox poise stoichiometry parameter between cytochrome b_6_f complex and photosystem II	NA
*b*_s_	The parameter controlling the speed of light-induced thylakoid swelling/shrinking with value given on the basis of absorbed photosynthetically active radiation	µmol^−1^ m^2^ s
*c*_s_	The parameter determining the maximum impact of macromolecular crowding on electron transport via the redox dynamics of cytochrome b_6_f complex	NA
Cyt	The cytochrome b_6_f complex	
*E*_T_	The composite temperature sensitivity parameter in the standardized temperature response function of electron transport derived from the Marcus theory, related to free energy of activation	K
ETC	Electron transport chain	
*f*_s_	The light-induced thylakoid swelling and shrinking function	NA
*f*_q_	The redox poise balance function between cytochrome b_6_f complex and photosystem II	NA
*f*_T_	The standardized temperature response function of electron transport in proteins according to the Marcus theory	NA
*H*_Cyt_	Fraction of cytochrome b_6_f complex available for linear electron transport	NA
*J*_PSII_	The linear electron transport rate from photosystem II to I	µmol m^−2^ s^−1^
$J_{\mathrm{PSIImax}}^{\prime }$	The conditional maximum linear electron transport rate from photosystem II to I, which is the highest attainable linear electron transport rate when the thylakoid is not fully expanded	µmol m^−2^ s^−1^
$J_{\mathrm{PSIImax}}$	The intrinsic maximum linear electron transport rate from photosystem II to I, which is the highest attainable linear electron transport rate when the thylakoid is fully expanded	µmol m^−2^ s^−1^
NPQ	Nonphotochemical quenching	
*NPQ*	Variable for nonphotochemical quenching	NA
*N*_PSII_	The foliar concentrations of total photosystem II reaction centers per unit leaf area	µmol m^−2^
$N_{PQ_{T}}$	The foliar concentration of the free plastoquinone and plastoquinol pool per unit leaf area	µmol m^−2^
$N_{\mathrm{Cy}t_{T}}$	The total foliar concentration of the cytochrome b_6_f complex, including both uninhibited and inhibited complexes for linear electron transport per unit leaf area	µmol m^−2^
PAM	Pulse amplitude modulated	
*PPFD*	Photosynthetic photon flux density	µmol m^−2^ s^−1^
PC	Plastocyanin	
PQ	Free plastoquinone	
PQH_2_	Plastoquinol	
PSI	Photosystem I	
PSII	Photosystem II	
*Q*	Fraction of open photosystem reaction centers	NA
$q_{J_{\mathrm{PSIImax}}^{\prime }}$	The conditional optimal fraction of photosystem II reaction centers when $J_{\mathrm{PSIImax}}^{\prime }$ is achieved	NA
$q_{J_{\mathrm{PSIImax}}}$	The intrinsic optimal fraction of photosystem II reaction centers when $J_{\mathrm{PSIImax}}$ is achieved	NA
*q*_r_	The fraction of reversible photosystem II reaction centers	NA
Q_A_	The tightly bound plastoquinone	
Q_B_	The loosely bound plastoquinone	
*r*_d_ and *r*_r_	The second-order rate constant for the electron transfer from the reduced acceptor to PQ to form PQH_2_ and for the reverse reaction, respectively	m^2^*µ*mol^−1^ s^−1^
*R*_1_	The first resistance of electron transport = *r*_r_/*r*_d_	NA
*R*_2_	The second resistance of electron transport = *u*$N_{\mathrm{Cy}t_{T}}$*/r*_d_*N*_PSII_	NA
*T*	Leaf temperature	K
*T*_0_	Reference leaf temperature (=298.15 K)	K
*U*	The second-order rate constant for the oxidation of plastoquinol by the RieskeFeS protein of cytochrome b_6_f complex	m^2^*µ*mol^−1^ s^−1^
*U*	The maximum oxidation potential of free plastoquinone and plastoquinol by the cytochrome b_6_f complex = *u*$N_{PQ_{T}}N_{\mathrm{Cy}t_{T}}$	µmol m^−2^ s^−1^
*α*	Leaf absorptance in photosynthetically active radiation	NA
*β*	Fraction of leaf-absorbed energy allocated to PSII	NA
*Φ*_PSIImax_	Maximum photochemical yield of PSII	NA

Eq 1 shows that increasing *α* alone would lead to a proportional increase in *J*_PSII_, if other conditions remain the same. However, if the absorbed energy cannot be used effectively to drive electron transport and CO_2_ reduction, it may lead to an increase in NPQ or a decrease in *q*. Further, it may prevent light penetration into deeper canopies and therefore decrease the efficiency of vertical space use by plants, diminishing photosynthetic productivities in high-density cultivations on a per unit of ground area basis (Cutolo et al. 2023). This explains previous experimental findings that smaller sizes of photosystem light-harvesting antenna complex (i.e. decreased *α*) increased the productivity of tobacco plants (*Nicotiana tabacum*) (Kirst et al. 2017; Cardona et al. 2018; Leister 2023). These counterintuitive results illustrate the interconnectedness of different photosynthetic processes and importance of system consideration in efforts to improve photosynthetic productivity.

NPQ protects leaves under excessive light conditions by dissipating into harmless heat the absorbed energy exceeding the level needed by the biochemical reactions. This process can be slower than the fluctuation in sunlight intensity in natural environments. The slow relaxation of NPQ may siphon the energy from photochemistry and decrease photosynthesis when light intensity changes from high to low and photoprotection is no longer needed. Speeding up NPQ relaxation via bioengineering approaches can therefore potentially enhance photosynthesis in fluctuating light environments, as demonstrated in seminal studies of tobacco (*N. tabacum*) (Kromdijk et al. 2016) and soybean (*Glycine max*) (De Souza et al. 2022). However, accelerated relaxation of photoprotection was observed to reduce biomass accumulation in Arabidopsis (*Arabidopsis thaliana*) (Garcia-Molina and Leister 2020). This suggests that alteration of NPQ relaxation speed may cause concurrent changes in other compensating mechanisms and its net effect on photosynthesis may not be universal across species (Theeuwen et al. 2022). As shown in Eq 1, accelerated relaxation of photoprotection during the light transition from high to low can increase *J*_PSII_ only if the acceleration does not simultaneously decrease *q*. If *q* decreases and the ratio of $\frac{1+NPQ}{q}$ increases, *J*_PSII_ will decrease. Understanding how genetic modifications affect the dynamic relationship between *q* and *NPQ* will be key to finding the root cause of these inconsistent experimental findings across species.

Rubisco is an important target in the biochemical reactions for improving photosynthetic efficiency of crops. This enzyme catalyzes over 90% of the conversion of inorganic carbon to biomass globally, constitutes 30% to 50% of soluble proteins in plant leaves (Erb and Zarzycki 2018), and is present in essentially all (>99.5%) autotrophic organisms (Raven 2009). However, Rubisco can only turnover a few catalytic events per second and is thus not a particularly efficient enzyme. Further, it catalyzes both the carboxylation and oxygenation of RuBP (Bathellier et al. 2018). Bioengineering enhancement of the catalytic rate of Rubisco could potentially increase RuBP carboxylation and improve photosynthetic efficiency (Lin et al. 2014). Meanwhile, RuBP oxygenation could be reduced by bypassing photorespiration (Xin et al. 2015) or integrating C_4_ photosynthesis into C_3_ crops (Schuler et al. 2016; Ermakova et al. 2020). It has also been suggested that photosynthesis can be improved by altering carbon metabolisms to increase the production of desirable products such as starch and oil (Mitchell et al. 2020).

However, no advantages of modified photophysical or biochemical reactions could be fully and sustainably realized for photosynthesis in the field unless the photochemical reactions can support such modifications. Because the primary reactions of photosynthesis are mainly sequential, its rate is limited by the slowest link and depends on the energy supply–demand balance (Kramer and Evans 2011; Sharkey 2020). As the bridge between the photophysical (energy supply) and biochemical (energy demand) reactions, the photochemical reactions could be a bottleneck for improving photosynthetic efficiency. The ETC appears suboptimal for plant productivity under current environmental conditions. Previous studies demonstrated that enhancing the level of the cytochrome b_6_f complex (Cyt), a transit center within which mobile electron carriers (plastoquinone [PQ] and plastocyanin [PC]) exchange electrons, increased plant growth (Chida et al. 2007; Simkin et al. 2017; Ermakova et al. 2019). Proposed modifications of the photophysical or biochemical reactions may pose additional challenges for the performance of the ETC as higher *J*_PSII_ values will likely be required to realize the expected increase in the rate of CO_2_ assimilation. If the ETC cannot support an increased demand for electron transport, then energy balance may be disrupted, and photosynthesis may not be improved in the field. Further, the ETC may be overreduced. An overreduction of the ETC will lead to an increased production of reactive oxygen species, resulting in photodamages to the photosynthetic apparatus and decreasing photosynthesis (Sonoike 2011; Vass 2012). Whether improved photophysical and biochemical reactions can translate into a sustained enhancement of photosynthesis at the system level in the field will depend on the capacity and efficiency of ETC to deliver the increased rate of electron transport without risking increased photodamage due to overreduction of the ETC in fluctuating light environments.

The objective of this study is to develop a quantitative understanding of how the abundances of protein complexes and mobile electron carriers of the thylakoid and the redox reactions between them affect the capacity of electron transport and the degree of reduction of the ETC. With this understanding, I hope to gain insights on how the structural components of the ETC may be modified to boost the maximal rate of LET without risking an overreduction of the ETC and particularly the PSII which may lead to photodamage. As a corollary to this effort, I would like to illuminate the photochemical mechanisms underlying several previously unexplained experimental findings, which include that increased expression of Cyt improves photosynthesis and plant growth (Chida et al. 2007; Simkin et al. 2017; Ermakova et al. 2019), deficiency in PQ impairs photosynthesis (Cook and Miles 1992; Hunter et al. 2018), and increased PQ concentration boosted plant stress tolerance (Ksas et al. 2015). I derive photochemical equations that describe the intrinsic maximum electron transport rate and the corresponding reduction level of the ETC as functions of the kinetics of redox reactions between protein complexes and electron carriers. I then conduct sensitivity analyses to determine how the transport rate and reduction level are determined by the structural characteristics of the ETC based on data from a large collection of pulse-amplitude modulated (PAM) fluorometry and gas exchange measurements made on over 2 dozen species in different climates. Advances from this study will contribute to a system understanding of how the photosynthetic machinery can be optimized to sustainably increase photosynthesis in natural environments.

## Results and discussion

### The photochemical model of photosynthetic electron transport

The approach of this study relies on the photochemical relationship between *q* and *J*_PSII_, the 2 state variables of the ETC. This photochemical relationship is defined by the redox reactions between the protein complexes and mobile electron carriers of the ETC, which differs from but complements the photophysically defined Eq 1. As the light harvesting complexes capture photons and transfer excitation energy to the reaction centers and charge separation from the donors of reaction centers is initiated, some acceptors of the reaction centers accept electrons and become reduced. These acceptors are now unable to accept new electrons from the donors until they transfer their electrons to electron carriers in line (i.e. they are closed). Therefore, for electron transport to occur (*J*_PSII_ > 0), some reaction centers will have to be closed (*q* < 1). The photophysical Eq 1 predicts that *J*_PSII_ can be enhanced by increasing *q* for a given set of environmental conditions. But obviously, if *q* is at the maximum of 1 (no acceptors are occupied by electrons), there would be no electron transport from PSII down to PSI. Therefore, the *q*–*J*_PSII_ relationship defined by the redox reactions must be a peaked function. Where this peak is located for a given structure of the ETC is of importance to bioengineered optimization of electron transport. But before this peak can be determined, the following 2 basic questions concerning the state variables of the ETC need to be answered: for a given value of *J*_PSII_, what characteristics of the ETC control the value of *q*, and conversely, for a given value of *q*, what characteristics of the ETC control *J*_PSII_? Answers to these 2 questions are provided by the recently developed Open–Closed (OC) model of the states and redox reactions of complexes and electron carriers along the ETC (Gu et al. 2023).

According to the OC model, the following equation governs the photochemical relationship between *q* and *J*_PSII_ (Materials and methods):


(2) $$J_{\mathrm{PSII}}=\frac{2Uf_{T}\;f_{s}\;f_{q}(q_{r}-q)q}{(R_{1}+2R_{2}\;f_{s}\;f_{q}-1)q+q_{r}},$$

(3) $$U=uN_{PQ_{T}}N_{\mathrm{Cy}t_{T}},$$

(4) $$R_{1}=\frac{r_{r}}{r_{d}},$$

(5) $$R_{2}=\frac{u}{r_{d}}\times \frac{N_{\mathrm{Cy}t_{T}}}{N_{\mathrm{PSII}}},$$

(6) $$f_{T}=\sqrt{\frac{T_{0}}{T}}e^{E_{T}\left(\frac{1}{T_{0}}-\frac{1}{T}\right)},$$

(7) $$f_{s}=\frac{v}{v_{\max}}=\frac{1}{1+c_{s}e^{-b_{s}\times \alpha \mathrm{PPFD}}},$$

(8) $$f_{q}=\frac{1+a_{q}}{1+a_{q}\times q}.$$

Unlike the photophysical Eq 1, which is valid only for the lake model of photosynthetic unit connectivity, the form of photochemical Eq 2 is valid for any assumption regarding the connectivity of photosynthetic units (e.g. the lake or puddle assumption) although the precise values of its parameters may differ. Here, *U* is the maximum oxidation potential of the combined PQ/PQH_2_ pool by Cyt. $R_{1}$ and $R_{2}$ are interpreted as the first and second resistance of electron transport, respectively. *u* is the second-order rate constant for the oxidation of PQH_2_ by the RieskeFeS protein of Cyt. *r*_d_ and *r*_r_ are the second-order rate constants for the electron transfer from the reduced acceptor to PQ to form PQH_2_ and for the reverse reaction, respectively. $N_{\mathrm{PSII}}$, $N_{PQ_{T}}$, and $N_{\mathrm{Cy}t_{T}}$ are the total foliar concentrations of PSII, the combined PQ and PQH_2_ pool, and Cyt for LET, respectively. *q*_r_ is the fraction of reversible PSII reaction centers, which may be less than unity due to the presence of inhibited and Q_B_-nonreducing PSII reaction centers, and the 2-electron gate. Plant stress can lead to permanent inhibition of reaction centers (Porcar-Castell 2011). Q_B_-nonreducing reaction centers are those with a primary quinone electron acceptor Q_A_ whose reduction cannot lead to subsequent reduction of the secondary quinone acceptor Q_B_ and can only be re-oxidized by a back transfer of electron to the donor side (Lavergne 1982). The 2-electron gate refers to the need for Q_B_ to acquire 2 electrons from 2 singly reduced Q_A_s before becoming mobile (Stirbet et al. 2020).

Eq 2 also contains 3 function modifiers *f*_T_, *f*_s_, and *f*_q_. $f_{T}$ (Eq 6) is the standardized temperature (*T*) response function for modifying redox reactions. It is derived from the Marcus theory of electron transfer in proteins. *E*_T_ is a composite temperature sensitivity parameter related to the Gibbs free energy of activation. *T*_0_ = 298.15 K is the reference temperature. $f_{s}$ (Eq 7) is the light-induced thylakoid ultrastructure dynamic function quantifying the degree of thylakoid ultrastructural control on electron transport. This ultrastructural control is achieved via regulations on the impact of macromolecular crowding on the diffusion of mobile electron carriers and the effective availability of Cyt for LET (Gu et al. 2022). *v* is the total volume of thylakoid at a given level of PPFD and swells/shrinks in response to osmotic water fluxes into and out of lumen, similar to the guard cell turgor pressure dynamics. *v*_max_ is the maximum thylakoid volume when it is fully swollen. *b*_s_ controls the speed of light-induced swelling/shrinking whereas *c*_s_ inversely determines the maximum net impact of macromolecular crowding on the effective availability of Cyt for LET. *f*_s_ varies between a value determined by *c*_s_ (thylakoid shrunk to the minimum in the dark) and 1 (thylakoid maximally expanded in full light). $f_{q}$ is the photosynthetically controlled redox poise balance function between Cyt and PSII with *a*_q_ as a redox poise stoichiometry parameter. This function relates the fraction of Cyt available for LET, denoted by *h*_Cyt_, to the fraction of open PSII reaction centers (i.e. *q*) via *h*_Cyt_ = *f*_q_ × *q*. Materials and methods gives more details about the justification of *f*_T_, *f*_s_, and *f*_q_.

The OC model has been tested against a dataset that consists of simultaneous measurements of PAM fluorometry and gas exchange of light, CO_2_, O_2_, and temperature responses made on more than 2 dozen C_3_ and C_4_ species in Canada, China, The Netherlands, and USA, as described in detail in previous studies (Han et al. 2022; Gu et al. 2023). These species are distributed in climates ranging from boreal to the tropics. They include lianas, shrubs, boreal deciduous and evergreen needle-leaf trees, temperate deciduous trees, tropical deciduous and evergreen trees, C_3_ and C_4_ grasses, and crops. Table 2 summarizes the statistics of model parameters estimated in Gu et al. (2023) from species with measurements of joint light and CO_2_ response curves, which minimize the risk of overfitting. These parameters are used in the present study.

**Table 2.** The mean, median, minimum, maximum, standard deviation (SD), and coefficient of variation (CV) of composite photochemical parameters derived from species used in Gu et al. (2023) with measurements of both light and CO_2_ response curves and for which the intrinsic maximum LET rate ($J_{\mathrm{PSIImax}}$) and the corresponding intrinsic optimal fraction of open photosystem II reaction center ($q_{J_{\mathrm{PSIImax}}}$) can be reliably inferred. These statistics are calculated from the median of the replicates for each species. The lake model of photosynthetic unit collectivity is assumed

Parameter	Unit	Mean	Median	Minimum	Maximum	SD	CV
*U*	µmol m^−2^ s^−1^	1319	1105	602	2861	589	0.45
*R*_1_	Unitless	0.50	0.53	0.03	0.82	0.19	0.38
*R*_2_	Unitless	0.26	0.00	0.00	7.66	1.40	5.48
*q*_r_	Unitless	0.81	0.81	0.62	1.00	0.08	0.10
*E*_T_	K	19640	6246	1572	44862	22056	1.12
*a*_q_	Unitless	−0.57	−0.61	−0.86	−0.20	0.18	−0.32
*b*_s_	µmol^−1^ m^2^ s	0.00492	0.00412	0.00258	0.02726	0.00436	0.89
*c*_s_	Unitless	7.43	7.35	4.60	15.52	1.99	0.27

### The desired direction of genetic improvements of photosynthesis in the ETC state space

**Figure 1:**
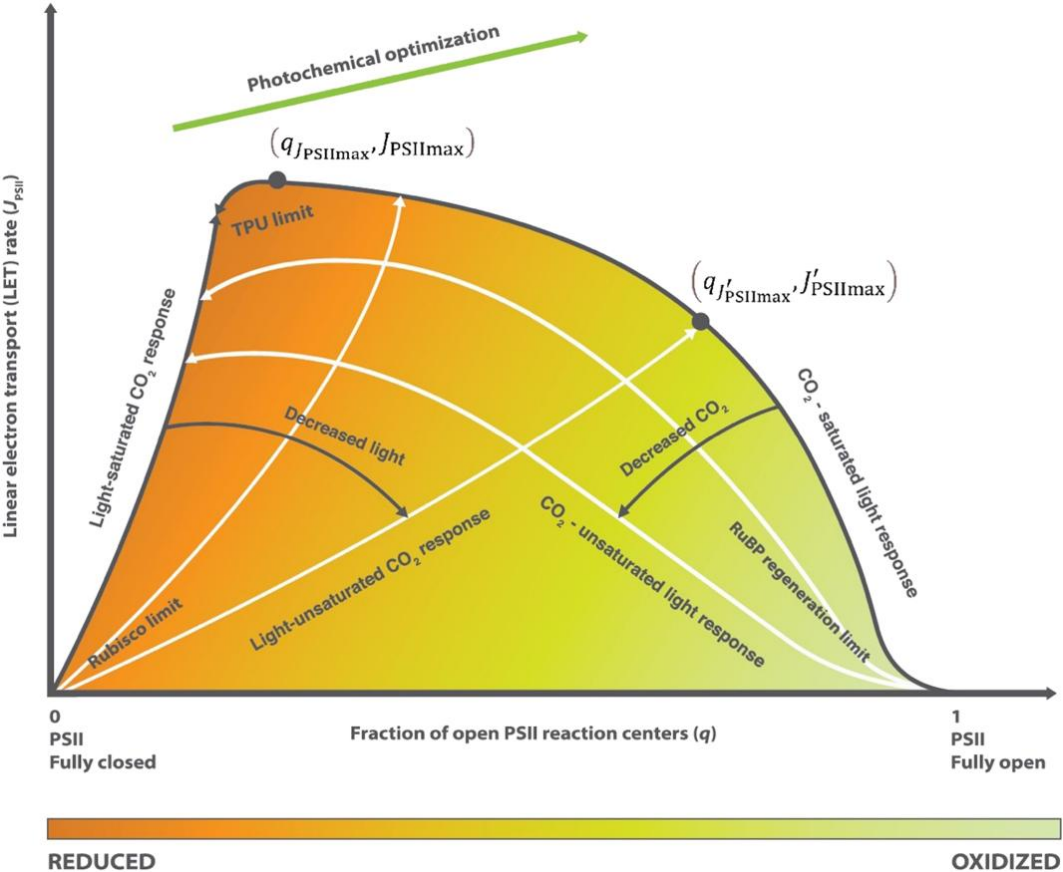
. The 2-dimensional state space of the photosynthetic ETC formed by the fraction of open PSII reaction centers (*q*) and the LET rate (*J*_PSII_). $\left(q_{J_{\mathrm{PSIImax}}^{{}^{\prime }}},J_{\mathrm{PSIImax}}^{\prime }\right)$ marks the intersection between a CO_2_ response curve at a subsaturating light level and the CO_2_-saturated light response curve, representing a conditional maximum *J*_PSII_ of a partially swollen thylakoid. $\left(q_{J_{\mathrm{PSIImax}}},J_{\mathrm{PSIImax}}\right)$ marks the intersection between a CO_2_ response curve at a saturating light level and the CO_2_-saturated light response curve, representing the intrinsic maximum *J*_PSII_ of a fully swollen thylakoid. Carboxylation limited by RuBP regeneration, Rubisco, and TPU each occupies 1 of the 3 corners of the *q*–*J*_PSII_ state space as marked. From right to left, the ETC is increasingly reduced as indicated by the shade gradient and at the leftmost, may be overly reduced, and risk photodamage due to oxidative stress. In general, bioengineered photochemical optimization can improve the efficiency of the electron transport without increasing oxidative stress by shifting the *q*–*J*_PSII_ state space toward the upper-right corner as indicated by the arrow at the top of the diagram.

Eq 2 depicts a 2-dimensional state space which defines how *q* and *J*_PSII_ covary in response to variations in environmental conditions as constrained by ETC properties (Fig. 1). For a given leaf, this state space can be established with typical PAM fluorometry measurements made with systematically varying ambient CO_2_ concentration and light intensity. For light response measurements made at a constant ambient CO_2_ concentration, *J*_PSII_ generally increases with a decrease in *q* (i.e. the reduction level of the ETC increases due to the growing supply of electrons) and peaks at some lower values of *q* as light intensity continues to increase. For CO_2_ response measurements made at a constant light level, *J*_PSII_ generally increases with an increase in *q* (i.e. the reduction level of the ETC decreases due to the growing demand of electron transport products in the biochemical reactions) and meets a light response curve at some higher value of *q* as the CO_2_ concentration continues to increase. For specific examples of these patterns, see Fig. 5 in Gu et al. (2023). The redox reactions of electron transport are affected by temperature (i.e. the *f*_T_ function in Eq 2). Also, the efficiency and capacity of the ETC depend on the degree of the ultrastructural control on electron transport which is determined by the light-induced swelling/shrinking of the thylakoid (i.e. the *f*_s_ function in Eq 2). Thus, the precise trajectory of the state variables (*q*, *J*_PSII_) within the *q*–*J*_PSII_ state space for a particular leaf depends on the temperature and light intensity. For a given temperature, the boundary of this space is formed by the light response with CO_2_ saturation at the high *q* side and the CO_2_ response with light saturation at the low *q* side. The *q*–*J*_PSII_ relationships made at subsaturating light or CO_2_ levels will fall below the CO_2_-saturated light or light-saturated CO_2_ responses. Carboxylation limited by RuBP regeneration, Rubisco, and triose phosphate utilization (TPU) occurs at the lower right corner, lower left corner, and upper left corner, respectively, of the *q*–*J*_PSII_ state space. The color gradient from right to left in Fig. 1 indicates that the ETC is increasingly reduced and, at the leftmost, may be overreduced with possible photodamage under stress. Ideally, successful optimization of the ETC for improving photosynthesis should increase the electron transport rate as much as possible and at the same time keep the degree of reduction of the ETC as low as possible to minimize potential photodamage. Diagrammatically, the desired direction of bioengineering efforts is to shift the *q*–*J*_PSII_ relationship towards the upper right corner of the ETC state space (higher maximum *J*_PSII_ at higher *q*).

### Structural and functional determinants of the maximum LET rate and corresponding reduction level of the ETC

To see how the desired direction of genetic improvements of photosynthesis in the ETC state space can be achieved, I investigate the photochemical determinants of the maximum *J*_PSII_ that can be supported by an ETC and the corresponding *q*. The maximum *J*_PSII_ can be located by finding the derivative of *J*_PSII_ with respect to *q* using Eq 2, setting it to 0, and solving the resulting equation for *q* (Fig. 1 and Materials and methods). This *q* is denoted by $q_{J_{\mathrm{PSIImax}}^{\prime }}$, which is given by the following:


(9) $$q_{J_{\mathrm{PSIImax}}^{\prime }}=\frac{q_{r}}{1+\sqrt{R_{1}(1+a_{q}q_{r})+2R_{2}\;f_{s}(1+a_{q})}}.$$

Inserting this $q_{J_{\mathrm{PSIImax}}^{\prime }}$ back into Eq 2, the corresponding maximum *J*_PSII_ (Materials and methods), denoted by $J_{\mathrm{PSIImax}}^{\prime }$, is obtained:


(10) $$\begin{array}{r} J_{\mathrm{PSIImax}}^{\prime }=\frac{2Uf_{T}f_{s}(1+a_{q})q_{r}}{R_{1}+1+a_{q}q_{r}+2R_{2}\;f_{s}(1+a_{q})+2\sqrt{R_{1}(1+a_{q}q_{r})+2R_{2}\;f_{s}(1+a_{q})}}. \end{array}$$

**Figure 2.**
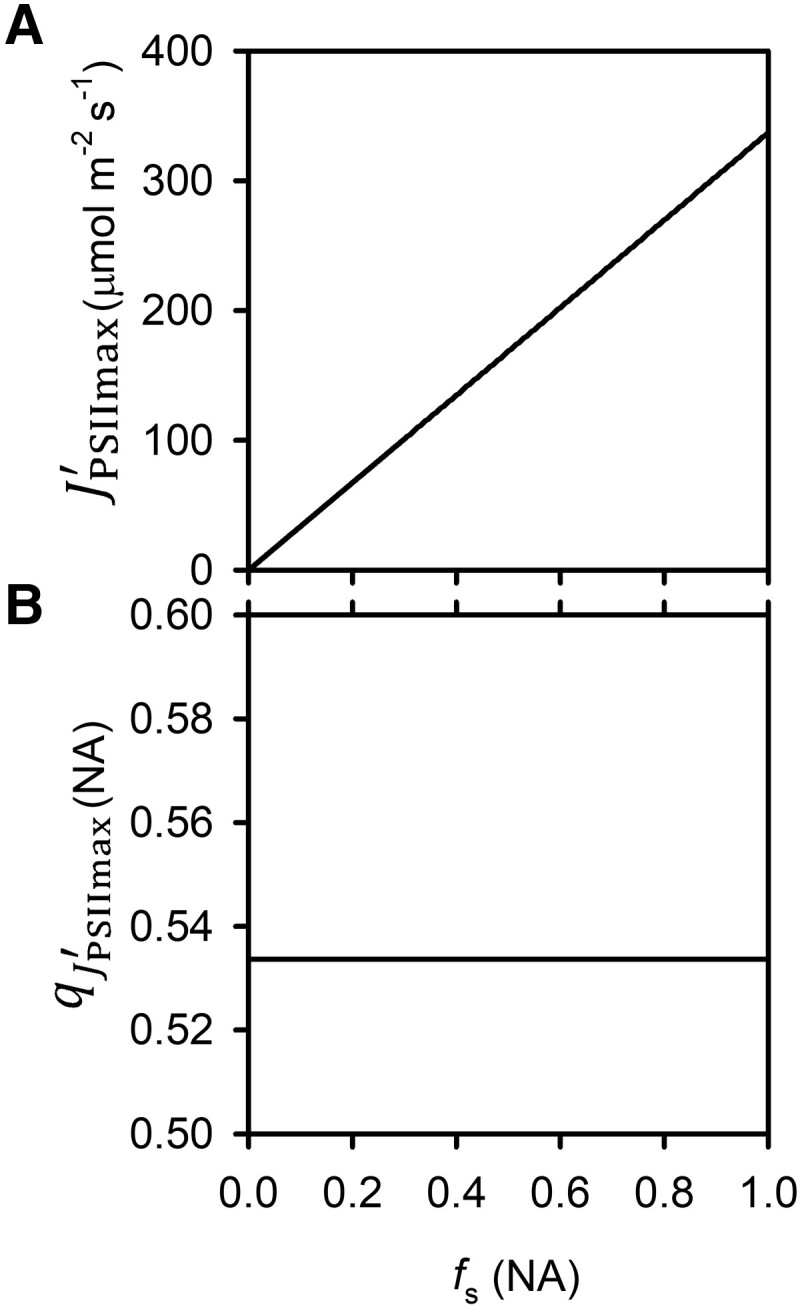
Variations of the conditional maximum LET rate ($J_{\mathrm{PSIImax}}^{{}^{\prime }}$) and the corresponding conditional optimal fraction of open PSII reaction centers ($q_{J_{\mathrm{PSIImax}}^{{}^{\prime }}}$) with the light-induced thylakoid ultrastructure dynamic function *f*_s_. The plots for $J_{\mathrm{PSIImax}}^{\prime }$**A)** and $q_{J_{\mathrm{PSIImax}}^{\prime }}$**B)** are created with the medians of the parameters given in Table 2. Both relationships appear linear because the estimated median $R_{2}$ is close to 0. For convenience, the figure plots *f*_s_ from 0 to 1. A 0 value of *f*_s_ is only a theoretical possibility, which would correspond to a thylakoid that is shrunk to such an extreme that macromolecular blocking is so pervasive that no electron carriers can move, which probably never happens in actuality.

In the *q*–*J*_PSII_ state space, $\left(q_{J_{\mathrm{PSIImax}}^{\prime }},J_{\mathrm{PSIImax}}^{\prime }\right)$ is the intersection between a *q*–*J*_PSII_ curve of CO_2_ response at a subsaturating light level and that of the light response at the saturating ambient CO_2_ level. It is interesting to note that temperature (i.e. the *f*_T_ function) does not affect the *q* value at which the maximum *J*_PSII_ is achieved even though it affects the latter. The thylakoid swelling/shrinking due to osmotic water influx and efflux (i.e. the *f*_s_ function) affects $J_{\mathrm{PSIImax}}^{\prime }$, which increases with *f*_s_ (i.e. when the thylakoid swells), as shown in Fig. 2A. Although *f*_s_ also appears in the equation for $q_{J_{\mathrm{PSIImax}}^{\prime }}$ (Eq 9), their relationship is muted in Fig. 2B. Figure 2 is produced with the medians of the photochemical parameters given in Table 2. Because the median *R*_2_ is close to 0 and also because *R*_2_ and *f*_s_ appear as a product in the denominators of Eq 9 and 10, $J_{\mathrm{PSIImax}}^{\prime }$ increases almost linearly with *f*_s_ whereas $q_{J_{\mathrm{PSIImax}}^{\prime }}$ appears insensitive to its variation in Fig. 2. The strong dependence of $J_{\mathrm{PSIImax}}^{\prime }$ on *f*_s_ indicates that macromolecular crowding is a major constraint on *J*_PSII_, which is alleviated by the light-induced thylakoid swelling.

$\left(q_{J_{\mathrm{PSIImax}}^{\prime }},J_{\mathrm{PSIImax}}^{\prime }\right)$ as described by Eq 9 and 10 is useful for discussing the maximum electron transport rate and the corresponding reduction level of the ETC for a given set of temperature and light level which interact with the structure of the thylakoid to determine photosynthetic electron transport capacity. From a bioengineering perspective, it is more convenient to focus on the structural constraints of the ETC. Environmental conditions affect $\left(q_{J_{\mathrm{PSIImax}}^{\prime }},J_{\mathrm{PSIImax}}^{\prime }\right)$ via the functions of *f*_T_ and *f*_s_. To remove the environmental impact, one can consider the special case of $\left(q_{J_{\mathrm{PSIImax}}^{\prime }},J_{\mathrm{PSIImax}}^{\prime }\right)$ at the standard condition of *f*_T_ = 1 (*T* = T_0_) and *f*_s_ = 1 (a fully swollen thylakoid). This special case is denoted by $\left(q_{J_{\mathrm{PSIImax}}},J_{\mathrm{PSIImax}}\right)$ as follows:


(11) $$q_{J_{\mathrm{PSIImax}}}=\frac{q_{r}}{1+\sqrt{R_{1}(1+a_{q}q_{r})+2R_{2}(1+a_{q})}}.$$

(12) $$\begin{array}{r} J_{\mathrm{PSIImax}}=\frac{2U(1+a_{q})q_{r}}{R_{1}+1+a_{q}q_{r}+2R_{2}(1+a_{q})+2\sqrt{R_{1}(1+a_{q}q_{r})+2R_{2}(1+a_{q})}}. \end{array}$$

**Figure 3.**
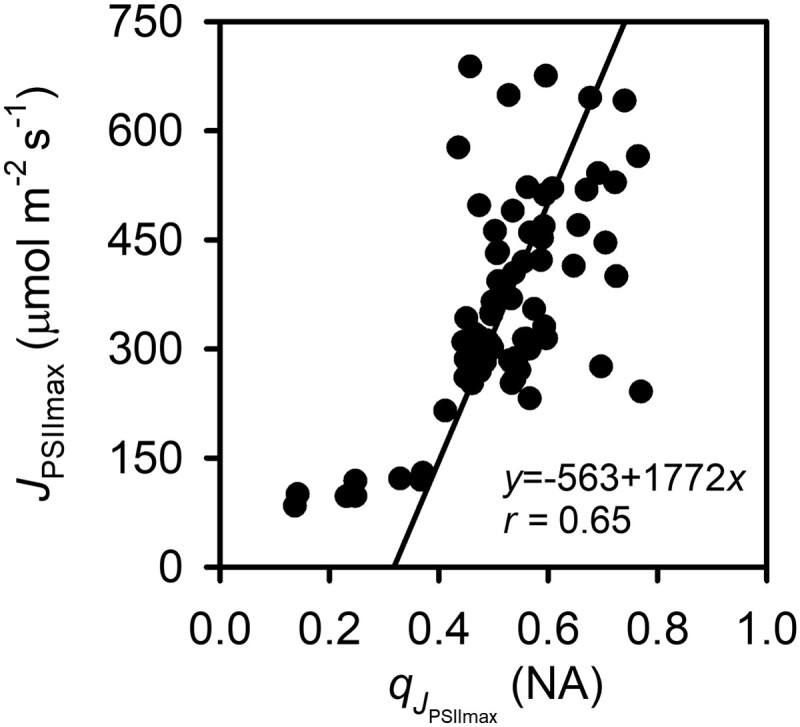
Variations of the intrinsic maximum LET rate (*J*_PSIImax_) with the corresponding intrinsic optimal fraction of open PSII reaction centers at which *J*_PSIImax_ occurs ($q_{J_{\mathrm{PSIImax}}}$), inferred from the species used in this study. The total least square linear regression with Pearson's coefficient was also given.

$\left(q_{J_{\mathrm{PSIImax}}},J_{\mathrm{PSIImax}}\right)$ is entirely determined by the structural characteristics of the ETC as represented by photochemical parameters *U*, *a*_q_, *R*_1_, *R*_2_, and *q*_r_. Both $q_{J_{\mathrm{PSIImax}}}$ and $J_{\mathrm{PSIImax}}$ are affected by *a*_q_, *R*_1_, *R*_2_, and *q*_r_ while *U* only affects $J_{\mathrm{PSIImax}}$ but not $q_{J_{\mathrm{PSIImax}}}$. $\left(q_{J_{\mathrm{PSIImax}}},J_{\mathrm{PSIImax}}\right)$ represents the peak formed by the *q*–*J*_PSII_ curve of CO_2_ response observed at the saturating light and that of light response observed at saturating ambient CO_2_ (Fig. 1). $J_{\mathrm{PSIImax}}^{\prime }$ is the highest attainable *J*_PSII_ when the thylakoid is not fully expanded (*f*_s_ < 1) whereas *J*_PSIImax_ is the highest attainable *J*_PSII_ when the thylakoid is fully swollen (*f*_s_ = 1). To differentiate $J_{\mathrm{PSIImax}}^{\prime }$ from $J_{\mathrm{PSIImax}}$, $J_{\mathrm{PSIImax}}^{\prime }$ is called the conditional maximum *J*_PSII_ and $J_{\mathrm{PSIImax}}$ the intrinsic maximum *J*_PSII_. The corresponding *q* values $q_{J_{\mathrm{PSIImax}}^{\prime }}$ and $q_{J_{\mathrm{PSIImax}}}$ are called the conditional and intrinsic optimal *q*, respectively. Across the C_3_ species for which joint light and CO_2_ responses were measured in Gu et al. (2023) and thus $q_{J_{\mathrm{PSIImax}}}$ and $J_{\mathrm{PSIImax}}$ can be reliably inferred, the inferred *J*_PSIImax_ increases with $q_{J_{\mathrm{PSIImax}}}$ (Fig. 3), indicating that the higher electron transport capacity is associated with more oxidized (less reduced) acceptors of PSII and reinforcing the desirable direction for bioengineering improvement of the ETC as shown in Fig. 1.

**Figure 4.**
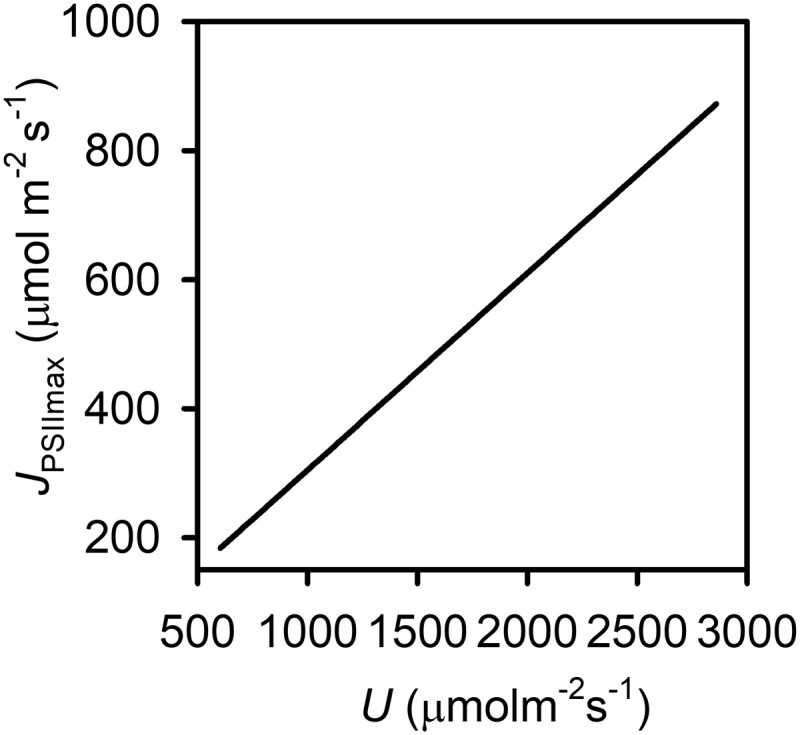
Variations of the intrinsic maximum LET rate (*J*_PSIImax_) with the maximum oxidation rate by Cyt $U=uN_{PQ_{T}}N_{\mathrm{Cy}t_{T}}$. This plot is created with the medians of the parameters given in Table 2 except for *U* which is plotted with its value between the minimum and maximum obtained in the dataset.

**Figure 5.**
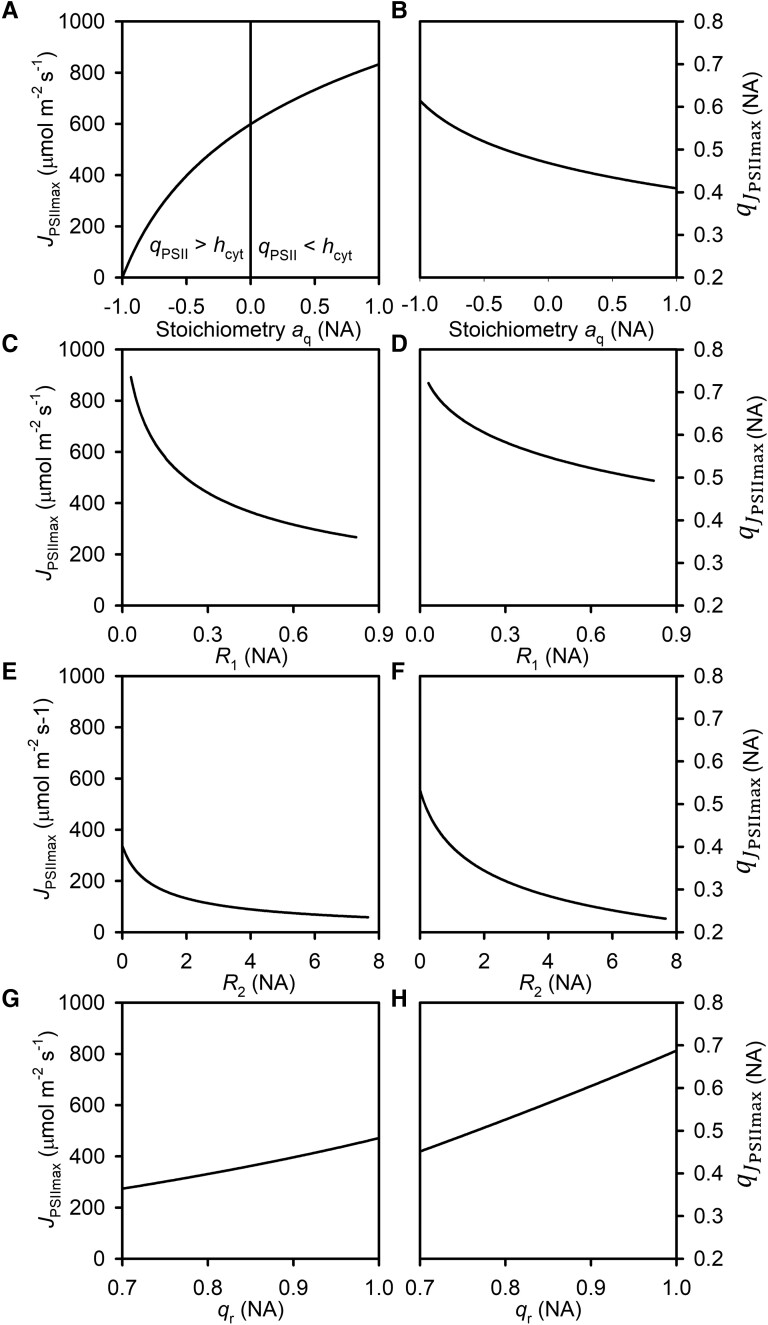
Variations of the intrinsic maximum LET rate (*J*_PSIImax_) and the corresponding intrinsic optimal fraction of open PSII reaction centers at which *J*_PSIImax_ occurs ($q_{J_{\mathrm{PSIImax}}}$) with key redox reaction parameters. **A, B)** The Cyt–PSII redox stoichiometry parameter *a*_q_. **C, D)** The first resistance of electron transport $R_{1}=\frac{r_{r}}{r_{d}}$. **E, F)** The second resistance of electron transport $R_{2}=\frac{u}{r_{d}}\times \frac{N_{\mathrm{Cy}t_{T}}}{N_{\mathrm{PSII}}}$. **G, H)** The fraction of reversible PSII reaction centers *q_r_*. These plots are created with the medians of the parameters given in Table 2, unless a parameter is systematically varied in a specific plot. The varied parameters are plotted with their values between their corresponding minimum and maximum obtained in the dataset. The only exception is *a*_q_ in Plots A and B which is plotted from −1 to 1 but has the obtained minimum and maximum of −0.86 and −0.20, respectively (Table 2). There is no a priori knowledge whether *a*_q_ should be negative or positive even though its maximum value obtained is negative. The range of *a*_q_ in Plots A and B is expanded to show the transition from a Cyt preferentially reduced (*a*_q_ < 0 and *q*_PSII_ > *h*_Cyt_) to PSII preferentially reduced (*a*_q_ > 0 and *q*_PSII_ < *h*_Cyt_) electron transport.

$J_{\mathrm{PSIImax}}$ is proportional to *U* (Eq 12; Fig. 4). It increases nonlinearly with an increase in *a*_q_ (Fig. 5A). The increase is sharper at low, negative *a*_q_ than at high, positive *a*_q_ values. In contrast, $q_{J_{\mathrm{PSIImax}}}$ decreases nonlinearly as *a*_q_ changes from negative to positive (Fig. 5B). $J_{\mathrm{PSIImax}}$ decreases nonlinearly with an increase in *R*_1_ with sharper decreases occurring at low than high *R*_1_ (Fig. 5C). The variation of $q_{J_{\mathrm{PSIImax}}}$ with *R*_1_ (Fig. 5D) is similar to that of $J_{\mathrm{PSIImax}}$. The relationships of $J_{\mathrm{PSIImax}}$ and $q_{J_{\mathrm{PSIImax}}}$ with *R*_2_ (Fig. 5, E and F, respectively) resemble their relationships with *R*_1_. Both $J_{\mathrm{PSIImax}}$ and $q_{J_{\mathrm{PSIImax}}}$ increase almost linearly with *q*_r_ (Fig. 5, G and H, respectively).

### Targets along the ETC for sustainable improvement of photosynthesis

The analyses above allow us to identify what and how components of the ETC can be modified to simultaneously increase the electron transport rate and minimize the degree of reduction of the ETC to control the risk of photodamage. $J_{\mathrm{PSIImax}}$ can be increased by increasing *U*, *a*_q_, and *q*_r_ or by decreasing *R*_1_ and *R*_2_ while $q_{J_{\mathrm{PSIImax}}}$ can be increased (i.e. the degree of ETC reduction can be decreased) by increasing *q*_r_ or by decreasing *a*_q_, *R*_1_, and *R*_2_. $U=uN_{PQ_{T}}N_{\mathrm{Cy}t_{T}}$ is the product of the rate constant for the oxidation of PQH_2_ by the RieskeFeS protein of Cyt and the abundances of the PQ and Cyt pools. The theoretically predicted increase of $J_{\mathrm{PSIImax}}$ with *U* (Fig. 4) explains the previous experimental findings that overexpressing the RieskeFeS protein of Cyt enhanced electron transport rates and biomass production (Chida et al. 2007; von Caemmerer and Furbank 2016; Simkin et al. 2017; Ermakova et al. 2019). Boosting RieskeFeS abundance essentially increases $N_{\mathrm{Cy}t_{T}}$ and therefore $U=uN_{PQ_{T}}N_{\mathrm{Cy}t_{T}}$. The biosynthesis of Cyt, a PQ–PC reductase, involves both chloroplast genes (Bruce and Malkin 1991) and nuclear genes (Voelker and Barkan 1995) and is also closely coregulated with that of other complexes such as ATP synthase (Schöttler et al. 2015) to ensure that energy supply is balanced with energy demand. Studies have found that Cyt has a long lifespan and appears to be synthesized primarily in young leaves and continuously functional as leaves age (Hojka et al. 2014). These characteristics of Cyt may allow its foliar abundance to be manipulated in multiple ways to increase the capacity of the ETC.

It is currently difficult to predict how the manipulation of foliar abundance of Cyt may affect the parameter *a*_q_ as the foliar abundance of PSII may also change due to potential coregulation in biosynthesis between these 2 complexes. If the abundances of both Cyt and PSII increase, then *a*_q_ may not change. However, if the abundance of Cyt increases relative to that of PSII, then there should be a decrease in the reducing pressure of electron transport on Cyt relative to that on PSII. This means that the Cyt–PSII stoichiometry parameter *a*_q_ should increase and *h*_Cyt_ may increasingly approach or even exceed *q* (Fig. 5A and Eq 8; also see Fig. 3 in Gu et al. 2023). Increased *a*_q_ can also lead to an increase in $J_{\mathrm{PSIImax}}$. A potential side effect is that $q_{J_{\mathrm{PSIImax}}}$ decreases with *a*_q_ (Fig. 5B). Thus, increasing *a*_q_ shifts the *q–J*_PSII_ state space to the upper-left corner, rather than the ideal upper-right corner, in Fig. 1, which may increase the risk of photodamage to PSII in fluctuating light environments. Future studies should investigate how bioengineered changes in Cyt may affect the reduction level of PSII to avoid unforeseen negative impacts.

Increasing the foliar abundance of PQ ($N_{PQ_{T}}$) can also increase *U* and therefore $J_{\mathrm{PSIImax}}$. Several previous studies support this model prediction. For example, sun leaves are photosynthetically more productive and have higher accumulation of PQ compared with shaded leaves (Lichtenthaler 2007; Szymańska and Kruk 2010). Photosynthesis is impaired in plants that are deficient in PQ. These plants suffer from pleiotropic effects and are incapable of photoautotrophic growth (Cook and Miles 1992; Hunter et al. 2018). Furthermore, increasing PQ abundance should lead to an increase in $q_{J_{\mathrm{PSIImax}}}$ because electron movement away from the acceptors of PSII is speeded up, protecting PSII from photodamage. This prediction is consistent with previous findings that high abundance of PQ boosts plant stress tolerance (Ksas et al. 2015). Therefore, genetically modifying plants to increase PQ abundance has the potential to shift $\left(q_{J_{\mathrm{PSIImax}}},J_{\mathrm{PSIImax}}\right)$ toward the desired direction in the *q*–*J*_PSII_ photochemical state space as shown in Fig. 1.

It is important to point out that electron transport between PSII and Cyt is only 1 of the many functions that PQ has in photosynthesis (Havaux 2020). The redox state of PQ regulates state transition that rebalances the energy absorption and allocation between PSII and PSI in response to environmental variations. It also serves as a redox sensor that regulates gene expression for the adjustment of stoichiometry and antenna sizes of photosystems (Escoubas et al. 1995; Allen et al. 2011). Other functions of PQ may include antioxidation, chloroplast metabolism, and biosynthesis of chloroplastic metabolites. Unlike Cyt, which has stable foliar concentration, PQ concentration can be very dynamic in response to variations in environmental conditions. Also, unlike Cyt, which is found only in the thylakoid membrane, PQ is present not just in the thylakoid membrane but also in the chloroplast envelope and plastoglobuli. Thus, efforts to increase PQ abundance should take into consideration its multifunctionality and multilocation characteristics.

The model presented in this study suggests that modifying redox reaction kinetics, if engineeringly possible, can also improve electron transport and photosynthetic capacity without risking increased photodamage. Faster oxidation of PQH_2_ by the RieskeFeS protein of Cyt (i.e. larger second-order rate constant *u*) can increase *U* and therefore $J_{\mathrm{PSIImax}}$. Improving the efficiency of the PQ reduction by the closed reaction centers with higher *r*_d_ and lower *r*_r_ to decrease *R*_1_ = $\frac{r_{r}}{r_{d}}$ and $R_{2}=\frac{u}{r_{d}}\times \frac{N_{\mathrm{Cy}t_{T}}}{N_{\mathrm{PSII}}}$ can simultaneously increase $J_{\mathrm{PSIImax}}$ and $q_{J_{\mathrm{PSIImax}}}$. *R*_2_ can also be decreased by increasing the foliar abundance of PSII reaction centers ($N_{\mathrm{PSII}}$) with reduced antenna size per reaction center. A great advantage of bioengineering efforts aiming at decreasing *R*_1_ and *R*_2_ is that they shift the state space of the ETC in the desirable direction that increases the electron transport capacity while simultaneously decreasing the risk of photodamage in fluctuating light environments. It may be productive to explore bioengineering of redox reaction kinetics along the ETC in conjunction with that of Rubisco.

Finally, increasing the value of *q*_r_, which can be achieved by decreasing the fraction of Q_B_-nonreducing PSII reaction centers, can also lead to an increase in both $J_{\mathrm{PSIImax}}$ and $q_{J_{\mathrm{PSIImax}}}$ (Fig. 5, G and H), shifting the *q*–*J*_PSII_ state space in the desirable direction for ETC bioengineering. A considerable fraction of PSII reaction centers may be Q_B_-nonreducing (Tomek et al. 2003; Schansker and Strasser 2005; Vredenberg et al. 2006; Vredenberg 2011). Currently, there is little knowledge regarding why such reaction centers exist at all and what their photochemical functions might be. Since the Q_B_-nonreducing fraction varies markedly across species and biotypes (Van Rensen and Vredenberg 2009), it may be possible to engineer crop species with fewer Q_B_-nonreducing centers. The median *q*_r_ for the species used in the present study is 0.81, indicating that there may be a substantive margin to increase it toward 1. Therefore, decreasing the fraction of Q_B_-nonreducing reaction centers may be an effective way to improving electron transport efficiency while keeping the reduction level of ETC as low as possible.

## Conclusion

The energized ETC is where major plant stresses are first sensed and photodamages first occur. Although light harvesting and CO_2_ assimilation have been the focused areas for bioengineering efforts to improve photosynthetic efficiency so far, whether these efforts can lead to sustainable enhancement of crop productivity depends on whether the ETC can support higher electron transport rates without increasing its degree of reduction. Diagrammatically, sustainable photosynthesis improvement requires shifting the photochemical *q*–*J*_PSII_ state space toward both higher *q* and higher *J*_PSII_ in Fig. 1. The present study shows that there are multiple ways to achieving this state space shift.

The regulation of photosynthetic electron transport is complex (Joliot and Johnson 2011; Gu et al. 2022). Nevertheless, the intrinsic maximal capacity $J_{\mathrm{PSIImax}}$ of the ETC for transporting electrons from PSII to PSI can be determined by as few as 5 composite photochemical parameters which are *U*, *a*_q_, *R*_1_, *R*_2_, and *q*_r_. Further, the degree of ETC reduction at which $J_{\mathrm{PSIImax}}$ occurs, which is inversely represented by the intrinsic optimal fraction of open PSII reaction centers $q_{J_{\mathrm{PSIImax}}}$, is determined by only 4 of the 5. Investigation of the compositions of these parameters and how they affect $q_{J_{\mathrm{PSIImax}}}$ and $J_{\mathrm{PSIImax}}$ leads to the identification of targets along the ETC that can be modified to shift the *q*–*J*_PSII_ state space in the desirable direction. Furthermore, these parameters can be estimated from typical PAM fluorometry and gas exchange measurements and conveniently employed to evaluate the potential for success of any bioengineering efforts to improve photosynthesis in field conditions.

The analyses of the present study are based on the OC photochemical model of electron transport, which is a steady-state model and assumes that the front end (the PSII portion) and the rear end (the PSI portion) of the ETC are in balance. This assumption enables the use of easily available data from PAM fluorometry to infer photochemical parameters of electron transport. It is carried over to the analyses conducted in this study. When the front end of the ETC is bioengineered for higher efficiency, it will be essential to make sure the rear end does not become a bottleneck for electron transport. For example, increasing the abundance of PSII or Cyt may also require a simultaneous increase in the abundance of PSI to realize an increase in $J_{\mathrm{PSIImax}}$ and $q_{J_{\mathrm{PSIImax}}}$. Likewise, improving the electron delivery efficiency of PQ between PSII and Cyt may also require a simultaneous increase in the efficiency of PC which transports electrons in the lumen between Cyt and PSI. A systems approach will be needed to ensure the proposed photochemical optimizations can lead to enhanced photosynthesis and plant growth in natural environments. Such an approach is greatly facilitated by coupling the photophysical model of Gu et al. (2019), the photochemical model of Gu et al. (2023), and the biochemical model of photosynthesis of Farquhar et al. (1980) and Sharkey (1985).

## Materials and methods

### The OC model of photosynthetic electron transport

The details of the OC model are described in Gu et al. (2023). Briefly, the model adopts the dichotomic representation of PSII reaction centers in PAM fluorometry and considers that a functionally reversible reaction center is in an either open or closed state. The donor of an open reaction center receives the energy of a photon and donates an electron to its acceptor, which closes the reaction center and initiates the chain of redox reactions. The redox reactions proceed with second-order rate constants. The PQ pool reacts with the pool of closed reaction centers and picks up protons from stroma. The reduced and protonated PQ (plastoquinol [PQH_2_]) is then oxidized by Cyt. These processes are represented by a system of differential equations. This system is then solved for the steady state to link the 2 state variables of the ETC (*q* and *J*_PSII_), which leads to Eq 2.

Three functions (*f*_T_, *f*_s_, and *f*_q_) modify the *q*–*J*_PSII_ relationship which is otherwise controlled by the physical structure of the ETC. *f*_T_ (Eq 6) represents the direct effect of temperature on rate constants and therefore electron transport during the photochemical reactions of photosynthesis. Temperature can also indirectly affect electron transport via downstream feedbacks from temperature-sensitive biochemical reactions, e.g. Rubisco activities as described in the widely applied Farquhar–von Caemmerer–Berry (FvCB) biochemical model of photosynthesis (Farquhar et al. 1980). Such feedbacks reflect demand for NADPH and ATP by the biochemical reactions and affect Cyt activities, which control the oxidation of plastoquinol, and the pH value in the lumen, which regulates NPQ. These biochemical feedbacks influence the overall response of the electron transport rate and therefore CO_2_ assimilation rate to environmental variations. They must be accounted for in a complete modeling framework of photosynthesis, which would include photophysics, photochemistry, and biochemistry (see Fig. 1 in Gu et al. 2023). The present study, however, focuses on the maximum electron transport capacity of the ETC as determined by its structural properties and does not deal with any realized rate of electron transport confined by this capacity in conjunction with biochemical feedbacks under a given set of environmental conditions. Therefore, only the direct effect of temperature on photochemistry is of relevance to the present study.

The ultrastructural control on photosynthetic electron transport and its representation by the *f*_s_ function (Eq 7) were based on several experimental observations which were discussed in detail elsewhere (Gu et al. 2022 and 2023). Only a summary is provided here. Thylakoid membranes are crowded with large protein complexes (e.g. light harvesting complexes, PSII/PSI reaction centers, and ATP synthase) with an estimated occupancy rate of 70% to 80% (Kirchhoff 2014a). These complexes can block the diffusion of PQ within the lipid bilayer cores for electron delivery from PSII to Cyt. Meanwhile, the lumen is narrow relative to the size of PC which is the lumen-confined electron carrier between Cyt and PSI. Also, the oxygen evolving complex (OEC) extrudes into the lumen. Thus, the diffusion of PC in the lumen can also be constricted. These barriers for the diffusion of PQ and PC can have strong impact on photosynthetic electron transport, particularly LET (Kirchhoff et al. 2011). This is because PSII and PSI are spatially segregated with PSII primarily in the grana stacks and PSI primarily in the stroma lamellae. LET cannot proceed unless the diffusion paths of electron carriers between PSII and PSI are unblocked. However, the thylakoid ultrastructure is rather dynamic. Electron microscopy has observed that thylakoid lumen swells in light and shrinks in the dark (Kirchhoff 2014b; Li et al. 2020). The swelling/shrinking is likely due to water fluxes into and out of lumen caused by water potential disequilibrium resulting from lumen acidification and ion movement across the thylakoid membrane (Beebo et al. 2013; Li et al. 2021). Photosynthetic electron transport is coupled with a buildup of transmembrane electric gradient, leading to ion movement via the ion channels in the thylakoid membrane toward the restoration of electroneutrality (Szabò and Spetea 2017; Geilfus 2018). Ion fluxes cause disequilibrium in water potential between the lumen and stroma and therefore osmotic water flow across the thylakoid membrane, which in turn swells the lumen. Model simulations have shown that the magnitude of swelling is sufficiently large to substantially facilitate electron transport in light (Höhner et al. 2020). The mechanism of thylakoid swelling/shrinking, although still needing further study, is perhaps analogous to the swelling/shrinking of guard cells that opens and closes stomatal pores. Gu et al. (2022) proposed a theory that suggests that land plants use thylakoid ultrastructural control to regulate electron transport in sync with gas exchange across stomata, which allows these species to survive and thrive in dry, high-irradiance environments. Gu et al. (2023) showed that the thylakoid swelling/shrinking must be explicitly represented in order to successfully model photosynthetic electron transport across a wide range of environment and species.

The basis for the Cyt–PSII redox poise balance function *f*_q_ (Eq 8) is that as more PSII are reduced, more Cyt should be reduced too. However, how exactly *h*_Cyt_ is related to *q* should be determined by the properties of the whole electron transport chain and feedbacks from biochemistry as quantified by the parameter *a*_q_. *a*_q_ = 0 gives the redox isocline between Cyt and PSII (*h*_Cyt_ = *q*; see Fig. 3 in Gu et al. 2023) and is a special case that has been assumed in previous studies (e.g. Johnson and Berry 2021). If *a*_q_ > 0, *h*_Cyt_ > *q*, indicating PSII is more strained than Cyt for LET. If *a*_q_ < 0, *h*_Cyt_ < *q*, indicating Cyt is more strained than PSII for LET. The *f*_q_ function provides a closure to the system of equations for modeling the photochemistry of photosynthetic electron transport. Analyses done by Gu et al. (2023) showed that *a*_q_ is consistently less than 0 and *h*_Cyt_ is substantially less than *q* across species, invalidating the previous assumption that the redox state of Cyt can be simply represented by the redox state of PSII.

### The derivation of $q_{J_{\mathrm{PSIImax}}^{\prime }}$ and $J_{\mathrm{PSIImax}}^{\prime }$

The derivation of $q_{J_{\mathrm{PSIImax}}^{\prime }}$ (Eq 9) and **$J_{\mathrm{PSIImax}}^{\prime }$** (Eq 10) starts by inserting Eq 8 into Eq 2 to obtain the following equation:


(13) $$J_{\mathrm{PSII}}=\frac{w(q_{r}-q)q}{xq^{2}+yq+q_{r}}.$$

Here,


(14) $$w=2Uf_{T}f_{s}(1+a_{q}).$$

(15) $$x=(R_{1}-1)a_{q}.$$

(16) $$y=R_{1}-1+q_{r}a_{q}+2R_{2}\;f_{s}(1+a_{q}).$$

The first-order derivative of *J*_PSII_ with respect to *q* is as follows:


(17) $$\frac{dJ_{\mathrm{PSII}}}{dq}=\frac{w(q_{r}-2q)}{xq^{2}+yq+q_{r}}-\frac{w(q_{r}-q)q(2xq+y)}{{\left(xq^{2}+yq+q_{r}\right)}^{2}}.$$

Setting $\frac{dJ_{\mathrm{PSII}}}{dq}$ to 0 and after some manipulations, one obtains the following:


(18) $$(xq_{r}+y)q^{2}+2q_{r}q-q_{r}^{2}=0.$$

$q_{J_{\mathrm{PSIImax}}^{\prime }}$ is the positive root of Eq 18, which is the following:


(19) $$q_{J_{\mathrm{PSIImax}}^{\prime }}=\frac{q_{r}}{1+\sqrt{1+xq_{r}+y}}.$$

Inserting Eq 15 and 16 into Eq 19 leads to Eq 9.

$J_{\mathrm{PSIImax}}^{\prime }$ is then found by inserting Eq 9 back into Eq 13:


(20) $$J_{\mathrm{PSIImax}}^{\prime }=\frac{w\left[q_{r}-\frac{q_{r}}{1+\sqrt{R_{1}(1+a_{q}q_{r})+2R_{2}\;f_{s}(1+a_{q})}}\right]\frac{q_{r}}{1+\sqrt{R_{1}(1+a_{q}q_{r})+2R_{2}\;f_{s}(1+a_{q})}}}{x{\left[\frac{q_{r}}{1+\sqrt{R_{1}(1+a_{q}q_{r})+2R_{2}\;f_{s}(1+a_{q})}}\right]}^{2}+\frac{yq_{r}}{1+\sqrt{R_{1}(1+a_{q}q_{r})+2R_{2}\;f_{s}(1+a_{q})}}+q_{r}}.$$

Eq 20 can be reduced to the following:


(21) $$J_{\mathrm{PSIImax}}^{\prime }=\frac{wq_{r}\sqrt{R_{1}(1+a_{q}q_{r})+2R_{2}\;f_{s}(1+a_{q})}}{xq_{r}+y+y\sqrt{R_{1}(1+a_{q}q_{r})+2R_{2}\;f_{s}(1+a_{q})}+{\left[1+\sqrt{R_{1}(1+a_{q}q_{r})+2R_{2}\;f_{s}(1+a_{q})}\right]}^{2}}.$$

Expanding the squared term in the denominator of Eq 21 and after some reorganization, the following equation is obtained:


(22) $$J_{\mathrm{PSIImax}}^{\prime }=\frac{wq_{r}\sqrt{R_{1}(1+a_{q}q_{r})+2R_{2}\;f_{s}(1+a_{q})}}{xq_{r}+y+1+R_{1}(1+a_{q}q_{r})+2R_{2}\;f_{s}(1+a_{q})+(y+2)\sqrt{R_{1}(1+a_{q}q_{r})+2R_{2}\;f_{s}(1+a_{q})}}.$$

Replacing *w*, *x*, and *y* in Eq 22 with Eq 14, 15, and 16, respectively, leads to the following:


(23) $$J_{\mathrm{PSIImax}}^{\prime }=\frac{2Uf_{T}f_{s}(1+a_{q})q_{r}\sqrt{R_{1}(1+a_{q}q_{r})+2R_{2}\;f_{s}(1+a_{q})}}{2[R_{1}(1+a_{q}q_{r})+2R_{2}\;f_{s}(1+a_{q})]+[R_{1}+1+q_{r}a_{q}+2R_{2}\;f_{s}(1+a_{q})]\sqrt{R_{1}(1+a_{q}q_{r})+2R_{2}\;f_{s}(1+a_{q})}}.$$

Dividing the nominator and denominator of Eq 23 by the common squared root term results in Eq 10.

## Data Availability

This study used previously published data of Gu et al. (2023).

## Dive Curated Terms

The following phenotypic, genotypic, and functional terms are of significance to the work described in this paper:

ATP CHEBI: CHEBI:15422

NADPH CHEBI: CHEBI:16474

r CHEBI: CHEBI:8735

electron CHEBI: CHEBI:10545
